# PI3K Mutation Profiles on Exons 9 (E545K and E542K) and 20 (H1047R) in Mexican Patients With HER-2 Overexpressed Breast Cancer and Its Relevance on Clinical–Pathological and Survival Biological Effects

**DOI:** 10.1155/2024/9058033

**Published:** 2024-10-15

**Authors:** T. Nieto-Coronel, Ortega-Gómez Alette, R. Yacab, E. A. Fernández-Figueroa, C. Lopez-Camarillo, L. Marchat, H. Astudillo-de la Vega, E. Ruiz-Garcia

**Affiliations:** ^1^Medical Oncology Unit, MyA Medic–Oncopalia Center, La Paz, Bolivia; ^2^Translational Medicine Laboratory, Instituto Nacional de Cancerología, Mexico City, Mexico; ^3^Core B of Innovation in Precision Medicine, National Institute of Genomic Medicine, Mexico City, Mexico; ^4^Posgrado en Ciencias Genómicas, Universidad Autónoma de la Ciudad de México, Mexico City, Mexico; ^5^Programa en Biomedicina Molecular y Red de Biotecnología, Instituto Politécnico Nacional, Mexico City, Mexico; ^6^Translational Research Laboratory in Cancer & Cellular Therapy, Hospital de Oncologia, Siglo XXI, IMSS-Instituto Mexicano del Seguridad Social, Mexico City, Mexico

**Keywords:** breast cancer, E454K, E542K, H1047R, HER-2 positive, PI3K mutations

## Abstract

**Background**: Trastuzumab resistance is associated with overexpressing the human epidermal growth factor receptor 2 (HER-2), which results from the altered phosphoinositide 3-kinase (PI3K) pathway in breast cancer patients.

**Objective**: We quantified the frequency of PI3K enzyme single and double-point mutations in Mexican patients with HER-2 overexpressing breast cancer and its association with clinical-pathological variables.

**Methods**: We embedded HER-2 breast samples in paraffin from 60 patients, extracted their DNA, and evaluated PI3K mutations in 49 HER-2-positive breast tumors. We focused on mutations for one exon 20 (H1047R) and two exon 9 PI3K (E545K, E542K) hotspots and characterized them as single and double-point mutations. The mean patient follow-up was 86 months.

**Results**: Of 49 patients who tested positive for HER-2 breast cancer, 14.28% showed mutations in PI3K, 71.42% single-point, and 28.56% double-point mutations. We found single-point mutations in H1047R (42.85%) and E545K (28.57%). Only two patients exhibited double-point mutations: one in E542K/E545K and another in H1047R/E545K (14.28% each). Although we observed lower survival in patients with mutations in PI3K, we did not find a significant association between these factors (*p* = 0.191). However, single and double-point mutations in PI3K were significantly associated with the clinical stages of diagnosis and tumor size (*p* = 0.027 and *p* = 0.04, respectively).

**Conclusion**: Single and double-point mutations in PI3K are related to tumor size and advanced clinical–pathological traits in Mexican patients with HER-2 overexpression, and future molecular studies are necessary to understand these findings.

## 1. Introduction

There were approximately 2.26 million breast cancer (BC) cases just in 2020, and the disease is the leading cause of cancer mortality in women worldwide, representing a pressing public health problem [1].

Although the preferred choice for BC characterization is molecular classification (i.e., estrogen receptor (ER), progesterone receptor (RP), and receptor of human epidermal growth factor 2 (HER-2), the classification based on pathological characteristics is still the most widely used method in clinical practice [2, 3]. However, molecular classification methods do not consider other relevant prognostic factors, such as tumor size and lymph node involvement.

**Figure 1:**
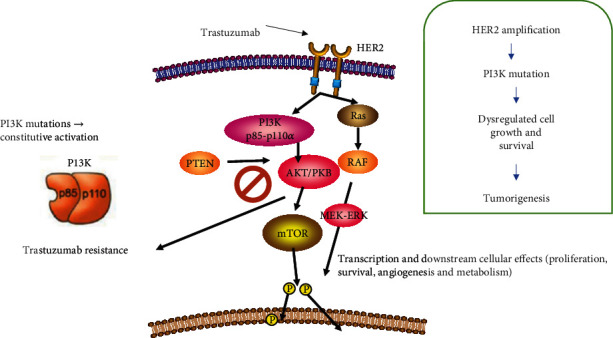
Role of the PI3K/Akt/mTOR signaling pathway in HER-2 mediated signal transduction in breast cancer. Inappropriate/excessive activation of the pathway caused by HER2 amplification, PIK3CA alteration, and/or PTEN loss-of-function can contribute to the formation of breast cancer via dysregulated cell growth, proliferation, and survival. mTOR: mammalian target of rapamycin, Akt: protein kinase B, HER: Human epidermal growth factor receptor, PI3K: phosphatidylinositol 3-kinase, P: phosphorylation, MEK: mitogen–activated protein kinase, ERK: extracellular signal–regulated kinase, PTEN, phosphatase and tensin homolog.

Patients with HER-2-positive BC under anti-HER-2 therapy usually have a short disease-free survival (DFS) and overall survival (OS) [4]. Despite anti-HER-2 therapy improving HER-2–positive BC patient's prognosis, primary and acquired resistance to anti-HER-2 therapy has been described [3]. In this regard, several research groups have tried to elucidate the molecular mechanisms of resistance to anti-HER-2 therapy [5], HER-2 is a transmembrane tyrosine kinase receptor, activated in a ligand-dependent or independent manner. HER-2 overexpression is considered an oncogenic driver that promotes constitutive activation of downstream signaling cascades related to cell survival/proliferation through MAPK pathway (Ras mitogen–activated protein kinase) or inhibition of cell death through PI3K/Akt/mTOR pathway [6]; trastuzumab resistance is associated with downstream signal activation, tumor stem cell self-renewal, host immune regulation, and epigenetic effects. HER-2 gene mutations modify the PI3K/AKT pathway, weakening the inhibitory effect of trastuzumab; furthermore, expression of other tyrosine kinase receptors and proteins in cell membranes may mask HER-2 receptors and prevent specific binding of trastuzumab to HER-2 through steric hindrance (Figure 1); these mechanisms have been recognized from “in vitro” studies, but their usefulness in clinical setting is uncertain [6]. In the neoadjuvant setting, a meta-analysis evaluating individual data from 967 patients with HER-2-positive BC included in five clinical trials evaluates the association of PI3K mutations with clinical outcomes, finding lower pathological complete response (pCR) rate in PI3K mutant compared with the wild-type (WT) group (16.2% vs. 29.6%, *p* < 0.001), with no statistically significant differences in terms of DFS and OS between groups; in the metastatic disease setting, at least two studies demonstrated significantly worse prognosis in patients with PI3K mutant tumors; biomarker analyses of two adjuvant trials (NSABP-B31 and FinHER) failed to identify any significant survival differences in patients with PI3K mutant or WT tumors; PI3K mutation showed a favorable prognostic impact in patients with PAM50 HER-2 enriched subtype in the adjuvant ShortHER trial. Data is clearly divergent about PI3K and HER-2 tumors [7].

PI3K is a family of serine/threonine protein kinases that belongs to the PI3K/AKT/mTOR pathway (phosphatidylinositol 3-kinase/protein kinase B/mammalian target of rapamycin). This pathway mediates physiological processes, including glucose homeostasis, protein synthesis, cell proliferation, survival, and angiogenesis (Figure 1) [8].

**Figure 2:**
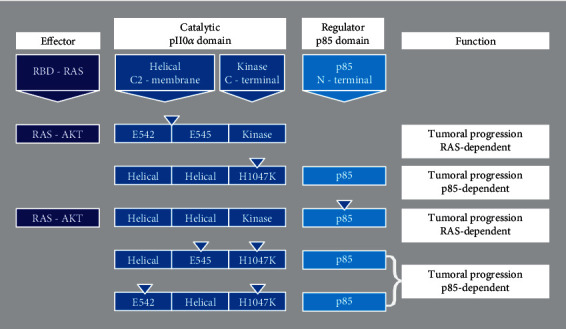
Mutational profiles on PI3K hotspots and tumor progression. Catalytic domain, p110*α* single mutations increase tumoral progression by helical domain-RAS-GTP and kinase domain-p85. Regulator p85 single mutations depend on RAS-AKT activity for tumoral progression. Double mutations (helical/kinase complex) induce gain-function by p85-AKT phosphorylation or by interactions with other subunits on p110*α* catalytic domain. RBD (RAS-binding Domain), AKT (Serine/Threonine kinase), RAS (small GTPasa). *Source:* Kalsi et al. [15]; Huang et al. [12]; Walker et al. [11].

The PI3K enzyme consists of two domains: the catalytic (p110) and the regulator domain (p85). Alternative splicing generates three isoforms of the catalytic subunit (p110): p110*β*, p110*γ*, and p110*α*. The latter is the most frequently mutated isoform in human tumors and is encoded by the PIK3CA gene [9]. The p110*α* subunit phosphorylates phosphatidylinositol-4,5-bisphosphate (PIP2) to generate phosphatidylinositol-3,4,5-trisphosphate (PIP3) in the plasmatic membrane [10]. P110*ɑ* is a complex subunit with several regions of interaction through the N-terminal domain, where it constitutively interacts with p85 (ABD (adapter-binding domain)) while interacting with the RAS protein through its carboxyl-terminal domain (Figure 2) [11, 12].

The p85 regulatory subunit stabilizes the p110*α* catalytic subunit and maintains it in a low activity state [13]. Growth factor stimulation and the subsequent activation of receptor tyrosine kinases (RTK) leads to autophosphorylation of tyrosine residues of the RTK, leading to PI3K activation, which is essential for controlling cell growth [14, 15]. The activation of the signaling pathways associated with the enzyme is related to the inorganic phosphoinositide 3-kinase pathway and the subsequent uncoupling of their subunits in the plasma membrane, where p110*α* activates RAS-GTP (the active form of GTPase RAS) through the RBD domain of p110*α* [16–18] (Figure 2).

There are activating mutations in PI3K in 20%–40% of the cases of BC [9], in both positive hormone receptors (Luminal A (45%) and Luminal B (29%)) and HER-2-positive BC (25%) [19, 20]. Mutations in the helical domain of exon 9 (E545K and E542K) and the kinase domain of exon 20 (H1047R) are the most frequently observed mutations [21, 22]; these mutations lead to amino acid changes that result in PI3K activity [23–25]. HER-2, a tyrosine kinase receptor, requires heterodimerization with HER-3 to activate the downstream signaling cascade. HER-3 has six p85-binding sites leading to activation of the PI3K pathway, whereas HER-2 lacks binding sites for the p85 subunit [26–29].

According to Reynoso-Noveron et al., 23.2% of Mexican women with BC have HER2-overexpression compared to the 25% described worldwide [30]. Because a quarter of the Mexican population with BC exhibits HER-2-positive tumors, it is crucial to know resistance mechanisms. This study describes the prevalence, type, and clinical outcomes of PI3K mutations in Mexican women with HER-2-positive BC.

## 2. Materials and Methods

### 2.1. Patient and Sample Selection

We obtained paraffin-embedded breast tumor samples from the Pathology Department at the Instituto Nacional de Cancerología, Mexico. This study has been approved by the ethics committee at Instituto Nacional de Cancerología (INCAN/of.CEI 592/13).

We identified 60 patients with BC and HER-2 overexpression by immunohistochemistry (IHQ) and FISH amplification. Fifty-nine patients were diagnosed with locally advanced BC stage and treated with anthracyclines, followed by paclitaxel and trastuzumab (anti-HER-2 therapy). After finalizing the treatment, patients were subject to surgery after evaluation by a surgeon who considered the clinical response to the treatment and the patient's approval. pCR was defined according to AJCC (American Joint Committee on Cancer) 8^th^ Edition as *yp*T0 and *yp*N0 when residual invasive cancer was not found in the breast tissue or lymph nodes. Eleven patients were excluded due to insufficient tumor tissue for DNA extraction.

We recorded biometric and clinical–pathological characteristics for each patient, including age at BC diagnosis, diabetes mellitus status or fasting glucose impairment, mean body index, lymph node involvement, and the estrogen and RP presence.

### 2.2. Analysis of HER-2 and Hormonal Receptors

ER, PR, and HER-2 status were evaluated by IHQ in the initially performed biopsy at Mexico's National Institute of Cancer (INCAN). A viable tissue sample was defined as the availability of a formalin-fixed paraffin–embedded (FFPE) breast tumor specimen with 70% or higher neoplastic cellularity. Samples were assessed by IHC according to the 2020 ASCO/CAP guidelines. The antibodies used were ER (clone SP1, Ventana, Arizona, United States), PR (clone 1E2, Ventana, Arizona, United States), and HER2 (4B5, Ventana, Arizona, United States). An independent batch of tumor tissues, processed in the same manner as our samples, was used for determining staining specificity. Serial titrations were performed in order to obtain optimal concentrations for every antibody. Additionally, antigen retrieval using Tris-EDTA or citrate was done. For chromogenic immunodetection, DAKO Envision systems or MACH 1 Universal HRP Polymer and diaminobenzidine were used. Afterwards, samples were counterstained with hematoxylin. All samples were reviewed at low magnification by a BC pathologist who was blinded to patient characteristics and outcomes.

FISH amplification was done on selected FFPE tissues and 2 *μ*m slices were obtained. Tissue pretreatment was done using a Vyasis kit. For HER-2 hybridization, the HER-2 neu probe was used with pathologist indications. Next, 20 nuclei were evaluated using Zeiss epifluorescence microscopy and ISIS software.

In our study, hormonal receptors were evaluated with the *H*-score scoring method. In accordance with ASCO/CAP guidelines, an *H*-score of 1 or more was considered a positive cut-off for ER or PR. For this purpose, we scored HER-2-positive samples as HER-2 0, HER-2 +++, HER-2++, and HER-2+ (scores of three, two, and one, respectively). HER-2 0 or HER-2+ was considered negative, and HER-2 +++ was considered positive. On the other hand, HER-2 < ++ samples were analyzed by FISH amplification; those with a ratio > 2.2 were considered HER-2 positive.

### 2.3. DNA Extraction and PI3K Mutation Detection

We cut paraffin-embedded tissue of BC tumors into 10-mm thick slices and placed them in 1.5 mL tubes. Then, we washed them twice with xylol at 65°C, mixed them, and centrifuged them for 2 min at 17,000 × g. We washed the slices with absolute ethanol and dried them for 10 min at 65°C. Following the manufacturer's instructions, we extracted the DNA using an All Prep DNA/RNA FFPE kit (Qiagen, Molecular Systems). We incubated the tissue slices in PKD buffer and proteinase K at 56°C for 15 min. Then, we incubated the samples on ice for 3 min, centrifuged them for 15 min at 20,000 × g, and discarded the supernatants. We incubated the resulting pellets in ATL buffer (Cat .80234 All Prep DNA/RNA FFPE Kit QIAGEN) and proteinase K for 1 h at 56°C and then for 2 h at 90°C. We added buffer and ethanol and mixed them by vortexing the samples. We transferred the supernatants to QIAamp MinElute spin columns. We centrifuged the tubes with the columns for 1 min at 8000 × g. We added buffer AW1 (Cat .80234 AllPrep DNA/RNA FFPE Kit QIAGEN) to each column, centrifuged them for 15 s at 8000 × g, discarded the supernatants, and added buffer AW2 (Cat.80234 AllPrep DNA/RNA FFPE Kit QIAGEN). We centrifuged the columns as described, added ethanol, and centrifuged. We placed the filtered content of columns in new tubes and centrifuged them for 5 min at full speed. We eluted the DNA using ATE buffer and quantified it in a Nanodrop 2000 device.

To analyze PI3K mutations (E545K, E542K, and H1047R), genomic DNA was processed according to the workflow of Custom qBiomarker Somatic Mutation PCR Array (QIAGEN; Cat. 337022; Custom ID CSMH02028RC). This PCR assay uses allele-specific primers designed to detect mutations or WT DNA. Allele-specific amplification is achieved by amplification refractory mutation system (ARMS) technology. The qualitative results are achieved by *C*_*T*_ values for each mutation-specific assay and the corresponding reference gene copy assay.

**Figure 3:**
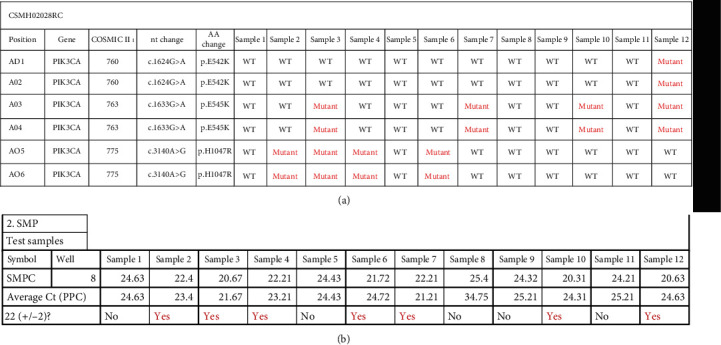
(a) qBiomarker somatic mutation PCR array. Qualitative analysis of PI3K E542K/E545K and H1047R mutations were evaluated. CT amplification data were calculated by subtracting the CT value (unknown sample) by the average values across samples with wild-sequence at the targeted locus (CT value = CT Test–CT Control). Wild samples of E542K/E545K and H1047R assays analyzed having CT values in the top 50 percentile assume the sample is a wild type for the assayed allele. When a CT test value is significantly smaller than the corresponding CT control value (CT test < <CT Control) by statistical analysis a positive mutation call can be made (average Ct (PPC)). (b) qBiomarker somatic mutation PCR array. Quantitative results of amplification data of each E542K/E545K and H1047R PI3K were obtained. CT amplification data were extracted from RT-PCR 7500 Fast Real Time PCR System. Average CT (PPC) analysis supports positive or negative mutation results.

Each gene is represented by its copy reference that measures DNA amount and quality; additionally, each array contains a normalizing copy number and positive PCR controls (SMPC) to test the presence of inhibitors or efficiency of PCR reaction using a predispensed artificial DNA sequence. The CT mutation value for each gene (CT mut) correlates to the total number of DNA copies positive for this specific mutation on catalytic or helicoidal domains. We show the data from qBiomarker Somatic Mutation Custom PCR Arrays (Figures 3(a) and 3(b)).

### 2.4. Statistical Analysis

We used the chi-square or Fisher's exact test to examine the relationship between clinical and histological variables of PI3K mutations. Statistically *p* value ≤ 0.05 indicates significant differences between mutated and nonmutated populations. For the survival analysis, we used a Kaplan–Meier test and the log-rank test for group comparison. We employed SPSS Statistics software V.23 for data analysis.

## 3. Results

### 3.1. Patient Characteristics

Our study included 49 patients with HER-2-positive BC tumors. We describe patient characteristics in Table 1. The mean age at BC diagnosis was 58.38 (40–75 years). Twelve percent of the patients had diabetes mellitus or impaired fasting glucose. The mean body mass index was 26.04 kg/m^2^ (18–38 kg/m^2^); 48.9% of the patients were overweight or obese, 38.8% of the patients had Stage II disease, 59.2% had Stage III, and just 2% had Stage IV disease. We found lymph node involvement in 85.7% of the patients. 36.7% and 26.5% tested positive for estrogen and RPs, respectively. HER-2 analysis was performed by IHQ in 76.7% of the patients, while 5% by FISH amplification.

All patients received anti-HER-2 therapy (trastuzumab) and taxane-based chemotherapy. Neoadjuvant chemotherapy was administered to 85.7% of the study population, with a pCR observed in 40.5% of patients, and 14.3% of the patients showed BC progression during neoadjuvant therapy.

### 3.2. Prevalence of PI3K Mutations

From the 49 HER-2-positive BC tumor samples, we found that 14.28% of the patients harbored a PI3K mutation; 71.42% had single mutations, and 28.58% had double mutations. Single H1047R mutation accounted for 42.85%of the mutations and single E545K mutation for 28.57%. Double mutation E542K/E545K and H1047R/E545K represent each 14.28% of the mutations (Table 2).

### 3.3. Clinical–Pathological Data of Patients Harboring PI3K Mutations

Of the seven patients exhibiting mutations, six showed a locally advanced disease. All patients received neoadjuvant therapy, but only three achieved pCR; two showed a single H1047R mutation, and one had a single E545K mutation (Table 3); after a mean follow-up of 86 months, they remained disease-free. However, patients who did not reach pCR died at 51 and 63 months. A patient harboring a double E545K/E542K mutation had a rapid disease progression while on neoadjuvant therapy and died after 11 months of follow-up. A 47-year-old patient with a triple-positive metastatic disease without visceral involvement who exhibited an E545K mutation died 8 months after starting the trastuzumab/paclitaxel treatment. Three patients, which means almost half (42.85%) of the patients harboring PI3K mutations also tested positive for hormone receptor expression, and one patient with a single E545K mutation had primary resistance to hormone therapy in her evolution (Table 3).

We found PI3K mutations associated with only two clinicopathological characteristics. PI3K mutation presence was associated with tumor size and clinical stage at diagnosis (*p* = 0.04 and *p* = 0.027, respectively, but if we omitted metastatic disease in the analyses and we did not find statistical significance (*p* = 0,738), we did not found relation with endocrine receptors presence (*p* = 0.909) nor with IHQ or FISH HER-2 positive (*p* = 0.466). Moreover, the rest of the clinical–pathological variables were not associated with PI3K mutation presence (Table 1).

### 3.4. Survival Analysis

While there was an observed difference in survival between the two groups (mutated versus no mutated), this difference did not reach statistical significance. Without mutations, they had a mean survival of up to 99.08 months; while with mutations, they did not survive past 67.85 months (*p* = 0.191 IC 95% HR = 2.97 [0.5–15.23]).

Our results do not have statistical significance but suggest that the type of mutation may change survival at a magnitude of two times. On average, patients with helical mutations survived 43 months, whereas patients with kinase mutations survived 93 months (*p* = 0.302). Furthermore, we did not find any significant relationship between survival and the presence of mutations (single or double) (*p* = 0.964).

## 4. Discussion

Most PI3K mutations reported in 25%–40% of BC patients occur in Exons 9 and 20 [23]. The frequency of PI3K mutations varies according to the BC subtype: In the Caucasian population, PI3K mutations are present in 34.5% of hormone receptor–positive patients, 22.7% of HER-2-positive patients, and 8.3% of basal–like BC patients harboring PI3K mutations [31]. In Latin America, 23% of Peruvian women exhibit HER-2–positive BC mutations, [32]. Nevertheless, the number of patients in our cohort was smaller than in the other studies.

There is controversy regarding which single mutation is the most common. Although research findings are mixed [20], it is generally accepted that the single E545K mutation is the most common [24, 33] since “in vitro” studies have demonstrated that it confers more significant PI3K activity [22, 34]. However, Bachman et al. showed that single Exon 20 mutations (H1047R) were more frequent [35], similar to the study by Castañeda et al. in Peruvian women with HER-2-positive and triple-negative BC [32]. In contrast, we found that the single mutation H1074 on exon 20 was the most prevalent in the Mexican women in our study with HER-2-positive BC. Studies demonstrate that Mexican ancestry heterogeneity is a consequence of mixtures of Native Mexican, European, and African populations using multiomics profiles. This heterogeneity observed in Mexican women with BC is influenced by mutations in the PI3K-AKT-mTOR signaling pathway [36].

Dependent and independent estrogen pathways regulate cell proliferation and survival in patients with positive hormone receptors. For example, the PI3K/AKT/mTOR pathway confers resistance to endocrine treatment [37, 38]. In our study, three patients with positive hormone receptors exhibited PI3K mutations. One of them also had primary endocrine resistance, and this data is not statistically significant.

pCR, defined as ypT ypN0, is associated with better OS in patients with HER-2-positive BC [39]. However, the role of PI3K mutations in this population is controversial since previous studies have not shown any statistically significant association between pCR, HER-2-positive, and PI3K mutations [31, 40]. There are studies in HER-2-positive tumors harboring PI3K mutations that suppress ERK and MEK phosphorylation mediating PI3K/AKT/mTOR signals and increasing its activity (mutations on kinase-helical domain) [41].

In our study population, there were three patients with mutations; 50% had pCR. However, these findings were not statistically significant. In future trials, the patient sample size could be increased.

Very few studies have focused on the impact of double mutations in BC, similar to the case of other cancer types (endometrium and colorectal) [42]. Hollestelle et al. found double mutations in 5% of BC cell lines, while Saal et al. identified double mutations (P539R/H1047R and E545K/K567) in 0.7% of patients with BC [42–44].

We identified two patients with double mutations (4.08%); one was present in the same helical domain; meanwhile, the other was in the kinase-helical domain; both patients had poor clinical outcomes. In contrast, Castañeda et al. did not find double mutations in a Peruvian cohort [30]. Kalsi et al. reported that double mutations in the helical domain (E545K and E542K) had less interaction with p85, altering its structure and function of PI3K leading to tumor proliferation [15]. Also, the coexistence of PI3K mutations in different domains seems to be associated with a worse prognosis; Zhao and Vogt, using chicken embryo fibroblasts, showed that double mutations of the kinase-helical domain (E545K/H1047R) triggered a gain of function in the p110*α* subunit, causing a synergistic effect and inducing the constitutive activity of PI3K [25, 42].

The relationship between the presence of mutations and survival is still controversial in BC [27, 28]. PI3K mutations are associated with improved DFS in colon cancer patients who received adjuvant therapy and improved radiosensitivity in patients undergoing yttrium 90 radioembolization [45, 46]. Our study showed differences with no statistical significance in OS between patients with and without mutations and single and double mutations.

Exon 20 single mutations (H1047R) were associated with better OS and progression-free survival of 163 patients with early BC. Exon 9 double mutations (E542K/E545K) were significantly associated with a worse prognosis [47]. The kinase domain (H1047R) may generate a conformational change of the PI3K loop triggering an oncogenic process [25]. Single H1047R mutations could also activate the catalytic domain of PI3K [24]. In xenograft models, the presence of PI3K mutations resulted in decreased cell proliferation and growth. The tumor mass was also reduced when cells expressed the H1047R mutation. This effect correlates with phosphorylation changes in downstream regulators such as AKT, ERK, and MEK, inducing transcriptional alterations in EGFR, ERBB2, and IGF-1R [41].

BC cell lines with mutations in the helical domain (Exon 9) have shown dependency on the 3-phosphoinositide-dependent-protein kinase 1 (PDK1) target for tumor growth. In contrast, BC cell lines driven by kinase domain (Exon 20) mutations are variable regarding their effects on AKT-phosphorylation and signaling pathways [48]. Although our study evaluated a limited number of patients, it seems that those cases with Exon 20 single mutations had better outcomes than those harboring Exon 9 single mutations.

The role of p85 in BC is still not clear. Thorpe et al. described that the homozygous deletion of phosphoinositide 3 kinase regulatory subunit 1 (PI3KR; gene encoding p85) is common in BC and results in p85 loss function, an increase in PI3K signalling and oncogenic transformation, thus, concluding that p85 had a role as a tumor suppressor [49]. While this study elucidates clinical connections between PI3K mutations and HER2-positive patients, it is crucial to highlight the necessity for future investigations into PI3K mutations linked to AKT-mTOR signals and therapeutic resistance. Further exploration in crosstalk between PI3K mutations and HER-2 significance requires more in-depth study. Mouse models have demonstrated that the combination of alpelisib (a PI3K inhibitor) with trastuzumab enhances apoptotic cell death in BC tumors. Moreover, utilizing a PI3K inhibitor in conjunction with trastuzumab in PI3K-mutated or PTEN-deficient HER2–positive BC cells results in increased apoptosis. To better understand this issue, additional experimental details are required, particularly pertaining to the 3D interactions between the intermutation and intramutation domains of the PI3K enzyme for clinical responses [50].

The role of PI3K mutation as a prognostic factor has been evaluated. A meta-analysis found that PI3K mutation was an independent negative prognostic factor (HR = 1.67, 95% CI 1.15–2.43, *p* = 0.007) [20], contrary to Buttita et al., who described a higher number of mutations in lobular versus ductal histology (40% vs. 20%, respectively); [44, 51] did not find a correlation between tumor histology and mutations. We described seven patients harboring mutations: Six had ductal, and one had lobular histology. There is still controversy about the association between PI3K mutations and tumor histology. Beelen evaluated the correlation between mutation status and histological grade in a cohort of 563 postmenopausal women with hormone receptor–positive BC. Single mutations in Exons 9 and 20 were associated with low-grade tumors [52]; this differed from Gershtein et al., who found a correlation between PI3K mutation and high-grade tumors [53]. Our findings demonstrate that patients with PI3K mutations had more large tumors and advanced disease.

Findings regarding PI3K mutations and lymph node involvement have been homogeneous; in a study by Saal et al., the presence of PI3K mutation was associated with lymph node involvement [44]; this agrees with our study, where 85% of patients with PI3K mutations had lymph node metastasis. We found a strong association between the PI3K mutations, larger tumor size, and advanced disease.

PI3K mutations are not routinely tested in HER-2-positive BC. Still, we can access this information by employing next-generation sequencing with variable results about PI3K mutations in the PCR kit. The evidence shows more aggressive and resistant HER-2-positive BC with PI3K mutations: In NeoALTTO [54], GeparSixto [55], and Neosphere [56] studies, PI3K mutations were linked to low rates of pCR and APHINITY study (adjuvant setting). PI3K/PTEN/AKT was associated with a poor prognosis, but KATHERINE [57] and ExteNET [58] showed no differences in adjuvant patients with or without PI3K mutations. In CLEOPATRA [59], THERESA [60], and EMILIA trials in the metastatic setting, PI3K mutation patients had shorter progression-free survival and OS. New therapeutical drugs combined with PI3K inhibitors and anti-HER-2 are now investigating. Evaluation of PI3K mutations will be essential in treating HER-2-positive disease BC [61].

The selective PI3K p110*α* inhibitor, alpelisib, approved by the FDA, demonstrated prolonged PFS among patients with the PIK3-mutant endocrine receptor positive in a metastatic setting in Phase III clinical trial SOLAR-1 [62].

Everolimus, an mTOR inhibitor, was evaluated in BOLERO (Breast Cancer Trials of Oral Everolimus). In BOLERO-1, everolimus combined with trastuzumab and paclitaxel as a first-line therapy for HER-2 positive in a metastasic setting (MBC) was tested against a placebo resulting in null benefits [63]. In BOLERO-3, previously treated women showing HER-2+ trastuzumab-resistant MBC were randomly assigned to everolimus or placebo in combination with vinorelbine and trastuzumab. Median PFS (progression-free survival) was 7.0 months in the everolimus group and 5.8 months in the placebo group (*p* = 0.006) but with toxicity issues [64]. BOLERO-1 and BOLERO-3 trials were conducted in a biomarker-unselected population of patients. Combining BOLERO-1 and BOLERO-3 trials suggested that patients with HER-2-postive MBC with aberrant PI3K pathway activation may have PFS benefits from everolimus [61].

Buparlisib is a panisoform Class I PI3K inhibitor with potent and selective activity against WT and mutant PI3K p110*α*. Buparlisib inhibits PI3K/AKT signaling in an ATP competitive manner decreasing PIP3 second messenger. In preclinical models of HER-2-positive BC, buparlisib has demonstrated significant antitumor activity, including synergism with anti-HER-2 treatments to revert trastuzumab resistance. Two clinical trials of buparlisib in HER-2+ disease show promising results. One of them, the PI3K HER-2 study (Phase IB), recruited patients with trastuzumab resistance, PI3K mutation–unselected HER-2–positive MBC. They were treated with buparlisib and lapatinib, resulting in a disease control rate of 79%, a clinical benefit rate (CBR) of 29%, and one patient with a complete response. However, the treatment also caused adverse events, including diarrhea, nausea, rash, depression, anxiety, increased transaminases, and asthenia [65].

NeoPHOEBE, a neoadjuvant trial program (Phase II), randomly assigned HER-2 positive patients (regardless of PI3K mutation status) to receive either buparlisib or placebo plus trastuzumab in the first 6 weeks, followed by buparlisib or placebo with trastuzumab and paclitaxel. The trial did not find significant differences in the pCR rate between the buparlisib and placebo arms (32% vs. 40%). Nonetheless, a near-significant trend was observed in the overall response rate (68.6% vs. 33%; *p* = 0.053) and a significant decrease in Ki67 levels (75% vs. 26.7%; *p* = 0.021) in the subgroup of patients with HR+/HER-2+ tumors. Patient recruitment was suspended mainly due to liver toxicity after the enrollment of 50 of the planned 256 patients [61, 66]. PI3KHER2 and NeoPHOEBE did not include PI3K mutations as a biomarker for selection, which may have affected the outcomes [61].

Phase I study of alpelisib and T-DM1 in patients with progressive disease on trastuzumab and taxanes provided new leads treating HER-2-positive MBC. The combination of alpelisib and T-DM1 is tolerable and demonstrated activity in trastuzumab–resistant HER-2-positive MBC. The results suggest that PI3K inhibition targets an important resistance pathway to anti-HER-2 therapy, providing the rationale for further study of PI3K inhibition in refractory HER-2-positive MBC to validate these results. This early trial did not select patients based on PI3K mutation status [61, 67].

Studies demonstrate that the heterogeneity of Mexican ancestry is a result of mixtures of Native Mexican, European, and African populations using multiomics profiles. This observed heterogeneity in Mexican women with BC is influenced by cancer genomes, including mutations in important pathways like the PI3K-AKT-mTOR signaling pathway [36].

This trial shows that PI3K mutations will be important in the future as a biomarker for HER-2-positive BC patients and shows for the first time PI3K mutations in BC patients in the Mexican population. But this trial has limitations: A small sample did not evaluate a broader spectrum of PI3K mutations and did not include functional analyses or in vitro studies for more comprehension about resistance in HER-2 patients with PI3K mutations. Further investigation of the molecular mechanisms and potential molecular targets of PI3K resistance in HER-2-positive BC is critical for improving therapeutic effectiveness and patient prognosis.

## 5. Conclusions

The prevalence of PI3K mutations in HER-2-positive BC patients in our study was lower than reported in the literature. The most common single mutation was H1047R, and double mutations were E545K/E542K and E545K/H1047R. We found a significant association between the presence of mutations with advanced stage and tumor size. Double mutations have a different biological behavior compared to single mutations. This behavior could be due to the interaction between other PI3K enzymatic domains, its conformational structure, or the activation of PI3K–independent signaling pathways. PI3K pathway and its clinical implication should be further investigated in HER-2-positive BC to improve patients' life expectancy.

## Figures and Tables

**Table 1:** General characteristics of the population (*N* = 49).

**Variable**	**Characteristics**	**N** ** (%)**	**PI3K mutations**		**p**
			**No**	**Yes**	
Age, years	≤ 50 years	9 (18.4)	8 (88.9)	1 (11.1)	0.619
	> 50 years	40 (81.6)	34 (85)	6 (15)	

Glycemia	DM 2	3 (6.1)	2 (66.7)	1 (33.3)	0.49
	Impaired glucose tolerance	3 (6.1)	3 (100)	0 (0)	
	Without modifications	43 (87.8)	37 (86)	6 (14)	

Nutritional status	Normopeso	23 (46.9)	20 (87)	3 (13)	0.57
	Overweight	10 (20.4)	9 (90)	1 (10)	
	Obesity G1	11 (22.4)	9 (81.8)	2 (18.2)	
	Obesity G2	3 (6.1)	3 (100)	0 (0)	
	Malnutrition	2 (4.1)	1 (50)	1 (50)	

Clinical stage at diagnosis	I	0 (0)	0 (0)	0 (0)	**0.027**
	II	18 (36.7)	17 (94.4)	1 (5.6)	
	III	30 (61.2)	25 (83.3)	5 (16.7)	
	IV	1 (2)	0 (0)	1 (100)	

Tumoral size (cm)	< 5.0	18 (36.7)	29 (69)	2 (28.6)	**0.04**
	≥ 5.0	31 (63.3)	13 (31)	5 (71.4)	

Lymph nodes	Negative	7 (14.3)	7 (100)	0 (0)	0.314
	Positive	42 (85.7)	35 (83.3)	7 (16.7)	

Metastasis	No	31 (63.3)	28 (90.3)	3 (9.7)	0.226
	Yes	18 (36.7)	14 (77.8)	4 (22.2)	

RE	Negative	31 (63.3)	27 (87.1)	4 (12.9)	0.71
	Positive	18 (36.7)	15 (83.3)	3 (16.7)	

RP	Negative	36 (73.5)	31 (86.1)	5 (13.9)	0.608
	Positive	13 (26.5)	11 (84.6)	2 (15.4)	

Abbreviations: DM2: type 2 diabetes mellitus; RE: estrogen receptors; RP: progesterone receptors; TNM: tumor nodes and metastases.

**Table 2:** Percentage of single and double mutation frequencies.

**Mutations**	**N** = 7** (14.28% of all population)**	**Type of mutation**	**Domain**	**Percentage (100%)**	**Percentage (100%)**
Single mutations	3	H1047R	Kinase	42.85	71.42%
	2	E545K	Helical	28.57	
Double mutations	1	E542K/545 K	Helical	14.28	28.56%
	1	H1047R/E545K	Kinase/Helical	14.28	

**Table 3:** Clinical characteristics of patients with PI3K mutations.

**Patients**	**Mutation**	**Age (years)**	**Comorbidities**	**BMI**	**CS**	**Histology**	**SBR**	**ER**	**PR**	**Treatment response**	**OS (months)**
1	E545K/H1047R	62	SAH	29.64	IIIA	IDC	7	70	240	PR	63
2	E542K/E545K	43	No	29.08	IIIA	IDC	7	0	0	DP	11
3	E545K	47	No	23.37	IV	IDC	7	60	0	DP	8
4	E545K	51	No	23.6	IIB	ILB		290	80	CR	110
5	H1047R	57	No	20.44	IIIA	IDC	8	0	0	CR	114
6	H1047R	57	DM2	29.86	IIIA	IDC	7	0	0	PR	51
7	H1047R	33	No	32.89	IIIA	IDC	8	0	0	CR	104

Abbreviations: CR: complete response; CS: clinical stage; DM2: type 2 diabetes mellitus; DP: disease progression; ER: estrogen receptor; IDC: invasive ductal carcinoma; ILB: invasive lobular breast cancer; OS: overall survival; PR: partial response; RP: progesterone receptor; SAH: systemic arterial hypertension; SBR: Scarff–Bloom–Richardson.

## Data Availability

The data used to support the findings of this study are included within the article.
